# Intrarenal Pressure Monitoring During Ureteroscopy: A Delphi Panel Consensus

**DOI:** 10.1016/j.euros.2025.01.005

**Published:** 2025-01-28

**Authors:** Bhaskar Somani, Niall Davis, Esteban Emiliani, Mehmet Ilker Göcke, Helene Jung, Etienne Xavier Keller, Arkadiusz Miernik, Silvia Proietti, Ben Turney, Oliver Wiseman, Antonia Bosworth Smith, Marco Caterino, Rhodri Saunders, Mohammed Boulmani, Olivier Traxer

**Affiliations:** a Department of Urology University Hospital Southampton Southampton UK; b Department of Urology Beaumont Hospital Dublin Ireland; c Department of Urology Fundación Puigvert Autonomous University of Barcelona Barcelona Spain; d Department of Urology Ankara University School of Medicine Ankara Turkey; e Department of Urology Hospital Lillebaelt Vejle Denmark; f Department of Urology University Hospital Zurich Zurich Switzerland; g Department of Urology University Medical Centre Freiburg Freiburg Germany; h Department of Urology San Raffaele Hospital Milan Italy; i Department of Urology University of Oxford Oxford UK; j Department of Urology Addenbrookes Hospital Cambridge UK; k Health Economics Coreva Scientific Königswinter Germany; l Urology and Pelvic Health Boston Scientific Paris France; m Groupe de Recherche Clinique sur la Lithiase Urinaire Sorbonne Université Paris France

**Keywords:** Consensus, Intrarenal pressure, Kidney stone, Ureteroscopy, Urolithiasis

## Abstract

**Background and objective:**

Elevated intrarenal pressure (IRP) may increase the risk of complications in patients undergoing ureteroscopy. As there is limited clarity on a threshold value for high IRP, how to manage high IRP, or which patients are at greater risk of complications due to high IRP, we used the Delphi methodology to understand expert opinion in this area.

**Methods:**

The Delphi process comprised two online surveys and an in-person meeting. During the in-person meeting, areas of disagreement and consensus were explored. Consensus statements were developed and voted on to determine the level of consensus. The study was granted a waiver by HML IRB Research and Ethics (reference number 2193).

**Key findings and limitations:**

The pan-European panel started with 12 and ended with 11 experienced endourologists. Eleven consensus statements were developed. The statements cover topics such as the definition of high IRP, complications linked to high IRP, and patient risk factors for these complications. After anonymous voting, consensus was achieved for all the statements. Two had a strong level and nine had a moderate level of agreement. There was no consensus on an IRP threshold, although the majority would be concerned for patient safety at a pressure above 61–80 cm H_2_O.

**Conclusions and clinical implications:**

Any IRP above normal physiological levels should be considered high. High IRP during ureteroscopy is a concern for patient safety. It is important to understand links between high IRP, patient characteristics, and complications. We call for additional research to better understand these risks and to inform refinements to clinical practice.

**Patient summary:**

A group of experts were asked their opinion on pressure within the kidney (intrarenal pressure, IRP) during a procedure called ureteroscopy (URS), when a narrow telescope is passed through the bladder and into the tube connected to the kidney. Statements that the panel agreed on were developed. These statements show that there is a concern about high IRP during URS as it may be linked to a higher risk of complications for the patient. More research is needed to better understand high IRP and its link to patient outcomes.

## Take Home Message

High intrarenal pressure (IRP) occurs during ureteroscopy and may put patients at risk of complications. Both research into IRP and monitoring of this parameter during procedures have potential to improve clinical practice and patient safety.

## 1 Introduction

The management of nephrolithiasis has seen a paradigm shift with the advent of minimally invasive surgeries [1]. Ureteroscopy (URS) has evolved significantly and is considered to be a first-line minimally invasive technique for the treatment of renal and ureteral stones, particularly those less than 20 mm in diameter [2].

There has recently been an increase in focus on intrarenal pressure (IRP) management during URS, with the need to achieve a balance between clear intraoperative vision, procedure time, and patient safety via adequate, but not excessive, irrigation. Flow imbalance arising from excessive irrigation inflow in relation to insufficient irrigation outflow can result in significant increases in IRP [3], [4]. This can potentially increase the risks of caliceal rupture, urosepsis, and renal damage [4], [5], [6], [7], [8].

Although ureteral access sheaths can help in reducing IRP [9], they do not obviate the need for continuous vigilance regarding IRP [10], [11]. Emerging technology for monitoring pressure presents surgeons with a potential avenue to augment their intraprocedural awareness of IRP and avert intraoperative and postoperative complications. A recent systematic review underscores growing consensus on control of IRP during URS to mitigate barotraumatic and septic complications [12]. Other studies have looked at the role of ureteral access sheaths and investigated locations for measuring IRP [13].

It has been reported that continuous IRP monitoring is a substantial contributor to reducing surgical and infectious complications [1], [5], [14], [15]. Despite the potential benefits of IRP monitoring, there is a lack of large-scale, high-quality studies determining its effectiveness. Thus, there is an absence of clear directives for validating and implementing IRP monitoring in clinical settings. Although recent recommendations from the International Alliance of Urolithiasis provide a clinical framework for urologists performing URS, there is not yet any guidance on IRP monitoring techniques, intervention thresholds, and eligibility criteria [2].

The aim of our study was to establish expert consensus on pertinent aspects of IRP and monitoring during URS via a Delphi process. Our focus was on exploring the potential benefits and risks of continuous IRP monitoring, and gaining an understanding of evidence gaps and obstacles in this field. The Delphi process relied on the clinical experience, expertise, and opinions of panel members to build on and extend knowledge from the literature. We hope that insights garnered from this process can help in identifying the direction of, and target groups for, future studies; in informing other urologists about how and when to use IRP monitoring; and in developing prospective guidelines for IRP monitoring.

## 2 Materials and methods

The primary aim of this Delphi study was to establish a consensus on IRP monitoring during ureteroscopic lithotripsy via an iterative, multistage process. The Delphi process does not have a defined number of panellists [16]; however, panels for health sciences should have between eight and 23 panellists [17]. Our panel included 11 experts, with participant numbers limited by the relatively small population meeting the criteria for an invitation to participate in the panel.

The panellists were selected from various European countries to obtain a pan-European perspective. Panellists were approached on the basis of their publication record on IRP and its measurement. All panellists were specialised endourologists and are currently practising, with an average of 15.9 yr of experience. The study was granted an exemption from ongoing review by HML IRB Research and Ethics (Washington, DC, USA; reference number 2193).

**Fig. 1:**
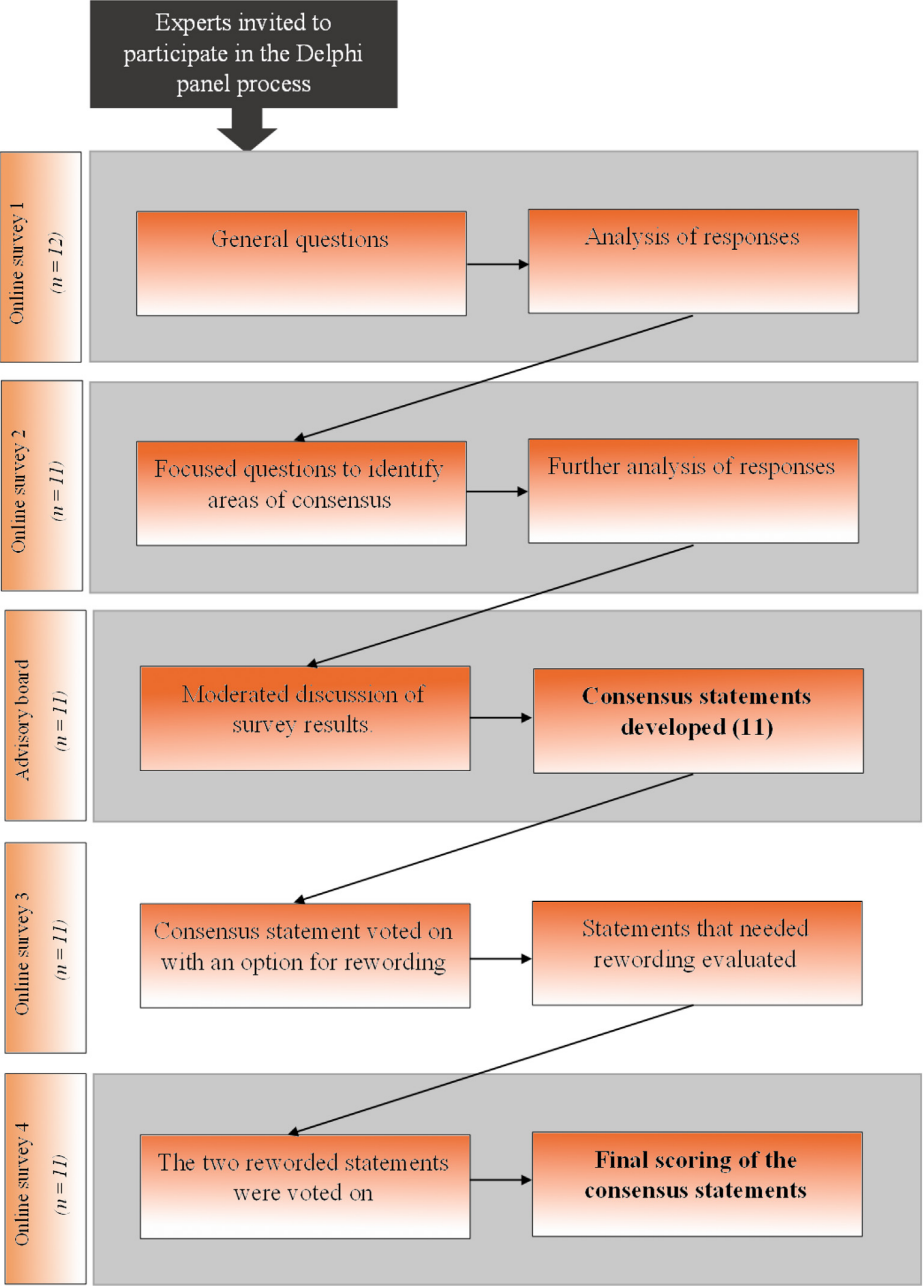
Flowchart for the Delphi process.

The Delphi process is outlined in Fig. 1. In summary, a mixed-methods Delphi process was undertaken that involved two online surveys to gather data and opinions before an in-person Delphi panel meeting for an in-depth discussion. Following the in-person meeting, voting on the consensus statements developed was undertaken anonymously online. Drawing from contemporary peer-reviewed literature, the first online survey was designed with mostly open-ended questions to understand the panel’s current thinking and opinions. Survey 1 also included some multiple-choice questions, Likert scales, and ranking tasks to capture the demographics, background, and experience with IRP monitoring for the panel members. All survey questions are included in the Supplementary material.

The second survey was devised using insights from the first questionnaire, with a focus on areas of contention: the threshold for high IRP of concern, related complications, recommended IRP measurement methods, and patient characteristics to consider.

Both online surveys had a 3-wk response window. Participants received comprehensive material about the survey’s aim, funding, and methodology, and could opt to withdraw throughout (as per IRB recommendations). The funding organisation, Boston Scientific, had no involvement in the study execution or interpretation of the results.

Responses were gathered anonymously and evaluated by two researchers independently. The consolidated findings were presented and discussed at an in-person meeting in Düsseldorf, Germany, in October 2023. Areas of potential consensus were shortlisted for debate and drafting of consensus statements. The consensus statements were derived from the opinions of the experts and were subsequently subject to an anonymised online voting process to quantify agreement within the panel. In the anonymous online scoring survey, each panel expert could select one of the following responses: “I fully agree” (scoring 3 points), “I partially agree” (scoring 2 points), or “I disagree” (scoring 1 point). If a panel expert selected “I partially agree”, they were asked to provide a better rewording of the consensus statement if they so wished. If more than 20% of participants partially agreed and suggested rewording, the consensus statement was voted on again, with variations based on any suggested rewordering included. Rewording and revoting on a consensus statement were done once at most.

**Fig. 2:**
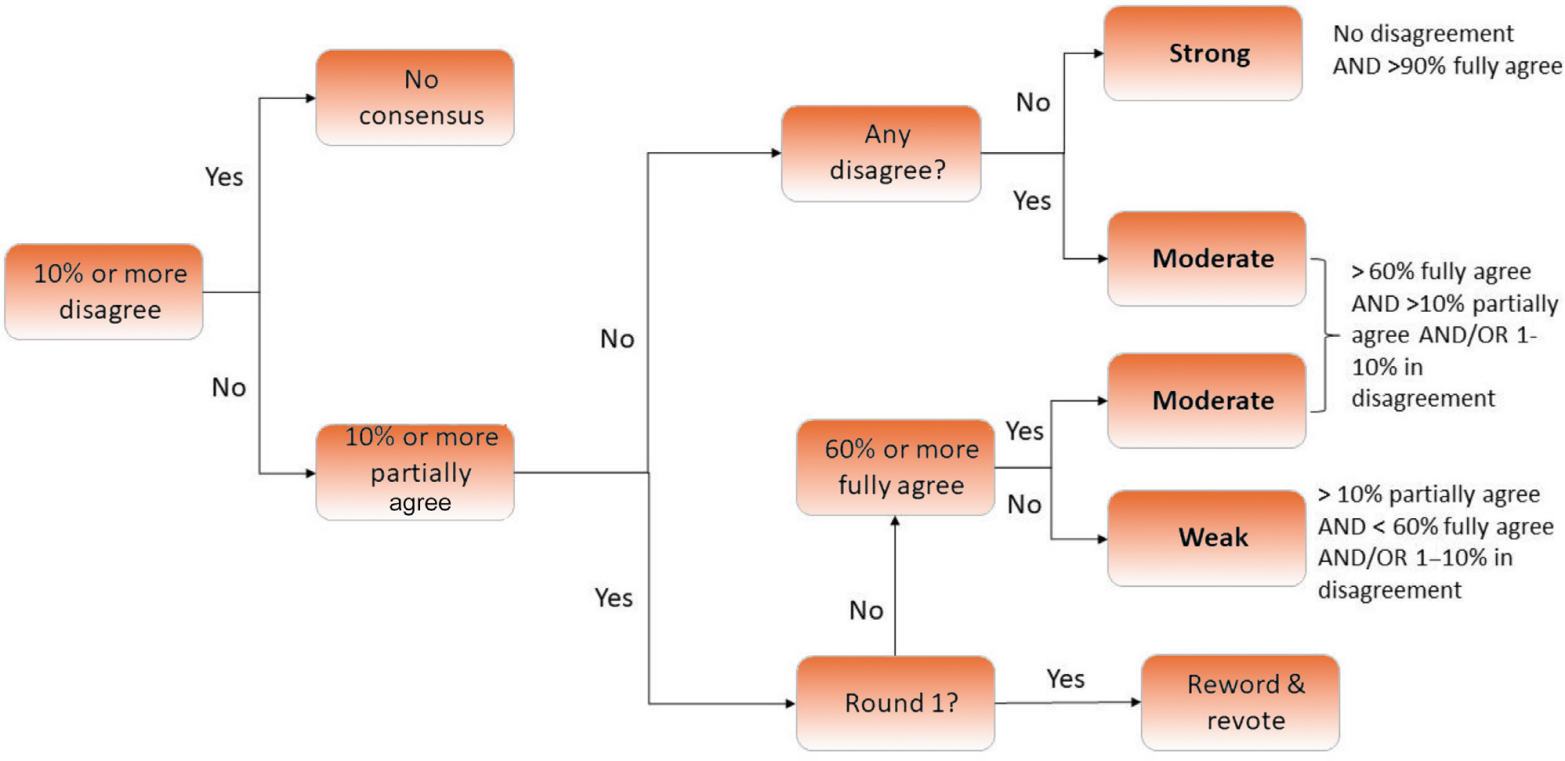
Decision tree for the strength of consensus statements.

When all the voting rounds were complete, each consensus statement was ranked as having strong, moderate, weak, or no consensus (Fig. 2) and its score was calculated to provide a hierarchy within the ranking system. This score was the sum of all responses given to the statement in its final round of voting, including any reworded versions if applicable.

During the Delphi process, definitions were important for a full understanding of the consensus statements. The following definitions were applied and agreed on during the in-person meeting:

­ Haematuria is a Clavien-Dindo grade ≥2 complication requiring additional intervention beyond standard practice.
­ Postoperative pain is a Clavien-Dindo grade ≥2 complication requiring additional analgesia beyond standard practice, an additional unplanned inpatient stay, or an additional intervention.

## 3 Results

### 3.1 The panel

Twelve endourology experts from Europe participated. Among these experts, nine were affiliated with university hospitals, two with public hospitals, and one with private hospitals. Their combined expertise covered the management of urolithiasis and prostate-related conditions. The median clinical tenure was 18 yr, with 46% of the participants in practice for more than 20 yr.

In terms of clinical metrics, experts reported a monthly median of 40 urolithiasis diagnoses and 30 stone removal procedures. The preferred surgical methods encompassed URS, percutaneous nephrolithotomy, and shockwave lithotripsy. Panel members also reported a monthly median of 20 URS procedures, with a strong preference for flexible URS (80%). Two of the experts reported active use of IRP monitoring during URS. All of the experts participated in the first survey phase. However, one opted out of the second phase, leading to 11 final participants.

### 3.2 Expert consensus

**Fig. 3:**
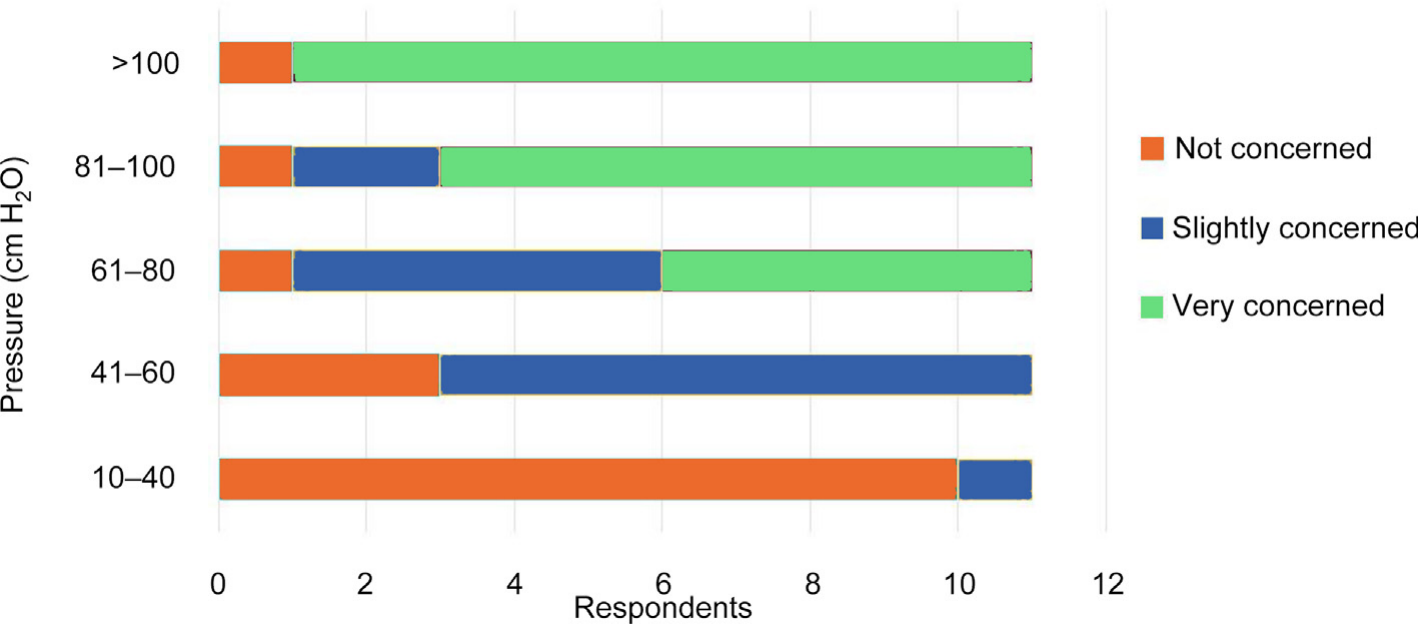
Results from survey 2 for the question “When is the pressure concerning?”.

The online surveys were used to inform and start the in-person discussion. One of the principal talking points was the point at which maximum IRP would be defined as concerning with a potential impact on patient safety (Fig. 3). In the survey, 10/11 participants reported being at least slightly concerned for IRP of 61–80 cm H_2_O and above. The survey did not provide an opportunity to discuss the reasons for this one differing opinion, but it was mentioned in the panel discussion that development of IRP is a multifactorial process, and more information would be helpful for determining the level of concern. In further discussions, no consensus was reached on a threshold value at which IRP might lead to an increase in the risk of adverse events. There was agreement that both IRP spikes and continued high IRP could lead to concern for patient safety, and even more so in patients with high risk of infectious complications.

Although definition of a precise “high” IRP threshold remained challenging because of limited data and prospective studies, there was unanimous recognition of the severe complications it might cause, including urosepsis, perirenal abscess, and death. Opinion was divided regarding the long-term outcomes of elevated IRP and the advantages of regular intraoperative monitoring.

Most experts identified no contraindication to IRP monitoring. However, cost considerations were prominently flagged as a potential barrier to widespread adoption. In addition, the panel highlighted a discernible need for standardised guidelines to facilitate interpretation of and response to IRP measurements. Nevertheless, the consensus leaned towards the fact that the current evidence is inconclusive and inadequate for recommending immediate changes in established clinical practices.

The Delphi process concluded with the formulation of 11 consensus statements that represent a synthesis of the collective expertise and insights of the panel. These consensus statements and their scores and rankings are presented in Table 1. In all 11 cases, consensus of at least a moderate level was achieved. Only two consensus statements, numbers 3 and 7 in Table 1, were reworded before being scored.

**Table 1:** Consensus statements on intrarenal pressure

Consensus statement		Score	Strength
1	High intrarenal pressure is a pressure that is above the normal physiological pressure in the kidney (circa 15 cm H_2_O).	32	Strong
2	The higher the intrarenal pressure, the higher the concern for patient safety. There is no consensus on a specific threshold. Initial concerns regarding intrarenal pressure start from 40 cm H_2_O. There is initial evidence that an intrarenal pressure over 100 cm H_2_O ± 50 cm H_2_O is of particular concern, but in some patients, values lower than this can also be of concern.	30	Moderate
3	Spikes and sustained high intrarenal pressure may both cause intrarenal pressure-related problems.	32	Moderate
4	Certain complications (see below) could arise in part due to high intrarenal pressure during the procedure, and it is advisable to work at the lowest pressure feasible to complete the procedure successfully. • Infection-related o Fever o UTI o Urosepsis • Postoperative pain • Bleeding related: o Intraoperative bleeding o Postoperative hematuria o Hematoma formation	31	Moderate
5	If there were no cost or resource constraints, we would recommend monitoring intrarenal pressure in all patients.	29	Moderate
6	Baseline and safe intraoperative renal pressure are individual to each patient. This could be linked to the patient’s characteristics.	32	Strong
7	Characteristics that could put patients at higher risk of complications from ureteroscopy could be broadly grouped into: • Infection o Recurrent UTIs o Infectious stones o History of positive urine culture o Immunosuppressed patients o Charlson comorbidity index score of ≥7 o Female o Diabetes o Prolonged ureteric stent dwell time • Procedure characteristics o Long procedure time • Anatomical conditions o Tight ureter o Narrow pelvic junction o Narrow infundibulum o Congenital anomalies o Ileal conduit	32	Moderate
8	There might be an association between intrarenal pressure and patient outcomes. Knowledge of intrarenal pressure could lead to changes in clinical practice. We need more data to investigate how intrarenal pressure monitoring is going to impact patient outcomes.	31	Moderate
9	More data are needed to investigate the association between intrarenal pressure and patient outcomes.	31	Moderate
10	Although rare, bleeding that impairs vision and leads to prolonged or aborted procedures could be linked to changes in intrarenal pressure during the procedure.	29	Moderate
11	Pressure readings may be clinically unreliable in particular situations, such as in the case of forniceal rupture.	31	Moderate

UTI = urinary tract infection.

## 4 Discussion

This Delphi study, which involved 11 endourology experts, has yielded insights into procedural IRP management, a domain of increasing clinical interest given recent technical developments that facilitate IRP monitoring. Elevated IRP raises concerns for patient safety and studies have considered associations between high IRP and adverse events, such as urinary tract infection, urosepsis, perirenal abscess formation, and, in extreme cases, mortality [18], [19], [20]. Setting a threshold for high IRP is complicated by the clinical reality of patient heterogeneity in physiology and comorbidities.

It is clear from our results that the point at which IRP becomes clinically relevant remains subjective and a notable area of disagreement among experts. There is uncertainty among experts regarding whether an absolute critical IRP value must be avoided, or whether the issue is more complex, with outcomes possibly being affected when IRP is raised above a lower level but for a sustained period of time. A further factor to consider is if the threshold should be a maximum value for spikes or for sustained pressure. These are practically different, but it was agreed that operating at the lowest possible pressure (sustained and spikes) is beneficial.

Although pressure in the kidney is normally low, the nature of URS means that IRP will increase. It could be surmised that working at a higher pressure can result in a shorter procedure time but may also result in larger spikes in pressure. The expert consensus was that high sustained pressure and or spikes in the pressure could negatively impact patient safety. Additional data on IRP and the spikes that occur during URS would yield greater insight into the procedure times spent at different pressures and the risk of complications. Given variations in anatomy and individual practise, there may be confounding factors to consider. For example, a study involving a silicone model to measure pressure during different procedures revealed that use of a ureteral access sheath and occupation of the working channel reduced the pressure [21].

According to the survey results, most experts would be concerned at pressures above 61–80 cm H_2_O (Fig. 3). The importance of IRP and the ability to measure it is clear, but an understanding of the clinical validity of these values and their implications requires further evaluation. Measurement of IRP per se will not directly improve patient safety; rather, it is the actions of the clinical team on identifying IRP of concern that will impact patient outcomes. IRP measurement should be a critical part of URS and can help in informing clinical decisions during the procedure. More research is required to indicate which clinical decisions should be informed by IRP, and how these decisions should be modulated by the information provided by IRP monitoring. Where feasible, it would be logical to measure IRP close to the target site. Currently there are no data to support the use of one method for IRP measurement over another. We recommend revisiting this issue once IRP measurement is more established.

The lack of consensus on a threshold value that should not be exceeded reflects the lack of data to support such a conclusion, as well as the inherent complexity of the procedure and individual variability for each operating surgeon’s technique, patient anatomy and physiology, and stone factors. Such diversity emphasises the need for dedicated studies to facilitate a more personalised, patient-centred approach to try and mitigate the risks associated with IRP during urological operations. These findings crystallise the collective expert concurrence on areas for which evidence remains sparse and further empirical research is required to delineate the exact ramifications of IRP monitoring for patient outcomes. This is a critical gap in contemporary urological research. The panel identified an urgent need for robust, structured, longitudinal studies with large cohorts that systematically measure and record IRP. Correlation of these measurements with clinical outcomes could generate important evidence in relation to IRP and patient outcomes to inform the development of more definitive and unified clinical guidelines and practice recommendations.

Intrarenal monitoring may be advantageous for patients; however, given the technological and resource constraints, monitoring is unlikely to be universally adopted. During this Delphi process, there was agreement on the patient characteristics that increase the risk of complications during URS. Although another similar statement regarding the link to IRP was developed, it scored slightly lower (score of 30, moderate agreement) and was not included. Following on from statement 7 (Table 1), these patient characteristics would be a natural starting point for selection of candidates for future IRP monitoring and clinical trials to refine the criteria for identifying those at risk of IRP-related complications. Preliminary clinical data suggest a tentative relationship between elevated and fluctuating IRP during URS and higher risk of fever and readmission [22]. A recent survey of 522 urologists showed a high level of concern related to elevated IRP, with 96% stating that they aim to reduce IRP during URS [23]. The potential adverse events the respondents associated with elevated IRP were urosepsis (96.2%), collecting system rupture (80.8%), postoperative pain (67%), bleeding (63.7%), and long-term renal damage (26.1%).

It was also highlighted that pressure readings may be unreliable in specific cases such as forniceal rupture or perirenal haematoma [24]. Although these events may frequently occur during URS, they remain rare causes of considerable irrigation fluid extravasation. Caution should be exercised regarding the irrigation pressure during URS irrespective of whether monitoring is available.

In a post hoc evaluation, the consensus statements were shown to 23 endourologists. Their voting results were similar to those of the Delphi panel (full findings in Supplementary Fig. 1). This similarity demonstrates that the opinions of the panel are generally in alignment with those of a larger group of urologists. Statements 2 and 3 (Table 1) had complete agreement. For statements 7 and 9 there was majority agreement, indicating that patient characteristics are important to the risk of complications and that further research is needed to fully understand the role of IRP.

Our study demonstrates some of the typical limitations of reliance on expert consensus as a primary source of insight. Sampling from a highly specialised and niche population, as was done here, limits the size of the sample. If grounded in extensive clinical experience, a Delphi process can bring practical ideas to the table, suggest how new technologies may impact current clinical practice, and determine which patients are most likely to benefit. However, it is difficult for expert panels to determine a numerical value for a threshold, for example, as this requires the rigour of data-driven research. The heterogeneity in opinions and consensus statements highlights areas particularly bereft of evidence, underscoring the need for robust, hypothesis-driven studies that could be partially informed by the consensus statements provided here. Prospective studies, ideally with diverse patient cohorts and multicentre designs, are needed to assess and quantify the impact of IRP and its monitoring on clinical outcomes and to establish more granular, patient-centred intervention thresholds and practice guidelines.

## 5 Conclusions

Consensus was reached for 11 statements on high IRP during URS and its monitoring. Strong consensus was reached for two statements, whereas moderate consensus was reached for the remaining nine statements. The two statements with strong consensus are statements 1 and 6, indicating that it is well accepted that IRP above physiological levels is high, and that patient characteristics have an important role in what a safe pressure reading would be. More clinical studies are required to better understand the risk of complications related to high IRP and the interplay of patient characteristics with these risks.

  ***Author contributions***: Bhaskar Somani had full access to all the data in the study and takes responsibility for the integrity of the data and the accuracy of the data analysis.

  *Study concept and design*: Saunders, Bosworth Smith, Caterino.

*Acquisition of data*: Saunders, Bosworth Smith, Caterino.

*Analysis and interpretation of data*: Davis, Emiliani, Göcke, Jung, Keller, Miernik, Proietti, Turney, Wiseman, Somani, Traxer.

*Drafting of the manuscript*: Somani, Caterino, Bosworth Smith, Saunders.

*Critical revision of the manuscript for important intellectual content*: Davis, Emiliani, Göcke, Jung, Keller, Miernik, Proietti, Turney, Wiseman, Boulmani, Traxer.

*Statistical analysis*: None.

*Obtaining funding*: Saunders, Boulmani.

*Administrative, technical, or material support*: Boulmani, Saunders, Bosworth Smith, Caterino.

*Supervision*: None.

*Other*: None.

  ***Financial disclosures:*** Bhaskar Somani certifies that all conflicts of interest, including specific financial interests and relationships and affiliations relevant to the subject matter or materials discussed in the manuscript (eg, employment/affiliation, grants or funding, consultancies, honoraria, stock ownership or options, expert testimony, royalties, or patents filed, received, or pending), are the following: Rhodri Saunders is the founder and Chief Executive Officer, and Marco Caterino and Antonia Bosworth Smith are employees of Coreva Scientific GmbH & Co KG, which received consultancy fees for performing, analysing, and communicating the work presented here. All of the remaining authors were part of the panel and were compensated by Boston Scientific for their time. Bhaskar Somani reports a paid consultant role for Boston Scientific, Pusen, Olympus, Cook Medical, and Coloplast. Ben Turney reports a consultant role for Boston Scientific, Pusen, and EMS, and research funding from Boston Scientific. Etienne Xavier Keller reports speaker and/or consultant roles for Boston Scientific, Coloplast, and Olympus, and funding from Boston Scientific for the present study. Niall Davis reports consultant and education roles for Boston Scientific. Mohammed Boulmani is an employee of Boston Scientific.

  ***Funding/Support and role of the sponsor*:** This study was funded by Boston Scientific. The sponsor played a role in review of the manuscript.
