# Supplementary material for: Intrarenal Pressure Monitoring During Ureteroscopy: A Delphi Panel Consensus
Source: Eur Urol Open Sci. 2025 Jan 28;73:43–50. doi: 10.1016/j.euros.2025.01.005 (PMC11815979; doi:10.1016/j.euros.2025.01.005)

Intrarenal Pressure Monitoring during Kidney Stone Removal: A Delphi Panel Consensus

Bhaskar Somani, Niall Davis, Esteban Emiliani, Ilker Göcke, Helene Jung, Etienne Xavier Keller, Arkadiusz Miernik, Silvia Proietti, Ben Turney, Oliver Wiseman, Antonia Bosworth Smith, Marco Caterino, Rhodri Saunders, Andreas Skolarikos, Mohammed Boulmani, Olivier Traxer

**Supplementary material**

# Survey 1

|  | Question | Answer type |
| --- | --- | --- |
| 1 | Which of these best describes your current workplace? | Multiple selection from university hospital, public hospital, private hospital, private practice, specialist clinic, and other |
| 2 | How would you describe your clinical specialty? | open, text |
| 3 | How many years of experience in your clinical specialty do you have? | open, n |
| 4 | On average, how many patients do you diagnose with kidney stones that need treatment each month? | open, n |
| 5 | On average, how many kidney stone removals do you perform in a month? | open, n |
| 6 | What method(s) do you use most frequently for kidney stone removal? | open |
| 7 | How many ureteroscopies do you perform a month? | open, n |
| 8 | How many of these are (in percentage) semi-rigid/flexible ureteroscopy? | n |
| 9 | What are the most frequent complications you encounter during ureteroscopic kidney stone removal? | open |
| 10 | What would you consider to be high intra-renal pressure? | open |
| 11 | In your direct experience, what is the incidence of high intra-renal pressure during ureteroscopy? | n (%) |
| 12 | Do you think high intra-renal pressure exposes patients to clinical complications? | open |
| 13 | Do you currently measure intra-renal pressure during ureteroscopy? | yes/no |
| 14 | If yes, in which patients do you measure intra-renal pressure?  If yes, how do you measure it? | open |
| 15 | If you were measuring intra-renal pressure, at which point would you:   - 1. Start becoming concerned about the pressure?   2. Stop the procedure due to high pressure? | n |
| 16 | In your opinion, what benefits could the introduction of intra-renal pressure monitoring during ureteroscopy bring to:   - - You:   - Your patients:   - Your clinical setting: | open |
| 17 | Which complications occur during ureteroscopic kidney stone removal?   - - In general   - Linked to high intra-renal pressure | open |
| 18 | Which are the most frequent complications linked to high intra-renal pressure during such procedures? | open |
| 19 | In your opinion, which and how many of these complications would be prevented with intra-renal pressure monitoring? | n, % |
| 20 | In your opinion, are there any long-term complications potentially linked to high intra-renal pressure during kidney stone removal? | open |
| 21 | Which patients have the greatest risk of experiencing high intra-renal pressure during ureteroscopy? | open |
| 22 | Which patients would be contraindicated for intra-renal pressure monitoring? | open |
| 23 | In your opinion, what percentage of patients undergoing kidney stone removal:   - - Could benefit from intra-renal pressure monitoring?   - Would it be contraindicated to receive intra-renal pressure monitoring? | open |
| 24 | In your opinion, how much evidence supports the use of intra-renal pressure monitoring during ureteroscopy? | open |
| 25 | In your opinion, is there adequate evidence supporting the use of intra-renal pressure monitoring during ureteroscopy? | yes/no |
| 26 | What, if any, are the primary evidence gaps? | open |
| 27 | Are there any patient or treatment factors that would deter you from recommending intra-renal pressure monitoring? | open |
| 28 | Do you have any concerns about the potential safety, resource, or training implications of introducing intra-renal pressure monitoring to your clinical setting? | open |

# Survey 2

|  | Question | Answer type |
| --- | --- | --- |
| 1 | The answers from survey one showed a wide range in terms of what would be considered high intra-renal pressure. Please indicate to what extent you would be concerned at each of the following intra-renal pressure ranges. | Rate the ranges |
| 2 | Assume that you are undertaking a procedure in which you are monitoring intra-renal pressure, which of the following would you use for clinical decision-making? | Single choice from list  *Absolute value, highest pressure reading*  *Sustained high pressure, average over 30 seconds*  *Sustained high pressure, average over 60 seconds* |
| 3 | In your opinion, do you agree or disagree that the following complications are increased due to high intra-renal pressure?  Bleeding  Damage from laser to the ureter and urothelium  Fever  Hematuria  Infection  Post-operative pain  Residual stones or stone fragments  Sepsis  Stent pain  Urosepsis | Strongly disagree – strongly agree |
| 4 | In your opinion, if you were to perform 1,000 ureteroscopies, how often would you expect to see the following complications?  Bleeding  Damage from laser to the ureter and urothelium  Fever  Hematuria  Infection  Post-operative pain  Residual stones or stone fragments  Sepsis  Stent pain  Urosepsis | n |
| 5 | Bleeding during ureteroscopy can impair the vision of the attending clinician. What percentage of your patient   1. Have bleeding that impairs your vision 2. Have bleeding that impairs your vision that extends procedure time 3. Have bleeding that impairs your vision that results in the procedure being cancelled | n |
| 6 | In your opinion, could bleeding, in some cases, during ureteroscopy be linked to high intra-renal pressure? | yes/no |
| 7 | In your opinion, do you agree or disagree that the following complications are increased due to high intra-renal pressure?   1. Chronic pain 2. Death 3. Hematoma formation 4. Peri-renal abscess 5. Potential renal impairment 6. Renal colic 7. Severe sepsis | Strongly disagree – strongly agree |
| 8 | In your opinion, if you were to perform 1,000 ureteroscopies, how often would you expect to see the following complications.   1. Chronic pain 2. Death 3. Hematoma formation 4. Peri-renal abscess 5. Potential renal impairment 6. Renal colic 7. Severe sepsis |  |
| 9 | In your opinion, which of these characteristics put patients at the highest risk for intra-operative complications? Please select your top 4, ranked from 1:highest risk to 4:lowest risk | Rank options |
| 10 | Is there any patient characteristic that would have been in your top four that was not included in the list above? | Open |
| 11 | Which of these patient characteristics are most important when determining who would receive intra-renal pressure monitoring? Please select your top 4, ranked from 1:most important to 4:least important. | Rank options |
| 12 | Are there any patient characteristics listed in the previous question that you think are irrelevant? If so, please explain. | Open |
| 13 | Do you agree or disagree that intra-renal pressure monitoring could prevent this complication? | Strongly disagree -Strongly agree |
| 14 | Below are the primary evidence gaps for intra-renal pressure monitoring identified in Survey 1. Please rank the top 4 most important, 1: most important to 4: least important. | Rank options |
| 15 | The following were barriers to intra-renal pressure monitoring use from you and your peers in Survey 1. Which are the top three that have the greatest impact on your practice? Please rank the top 4 that have the most impact on your practice. 1: most impact 4: least impact | Rank options |
| 16 | In terms of patient safety, please rank the following factors relative to intra-renal pressure monitoring. These factors were not mentioned in survey 1 but are taken from literature. | Less important than IRP/Equally as important as IRP/More important than IRP/Not relevant |
| 17 | If there is anything that we missed that you think is relevant, please include it here. This question is optional. | Open |

Supplementary Fig. 1 – Results of voting by the 23 external endourologists.


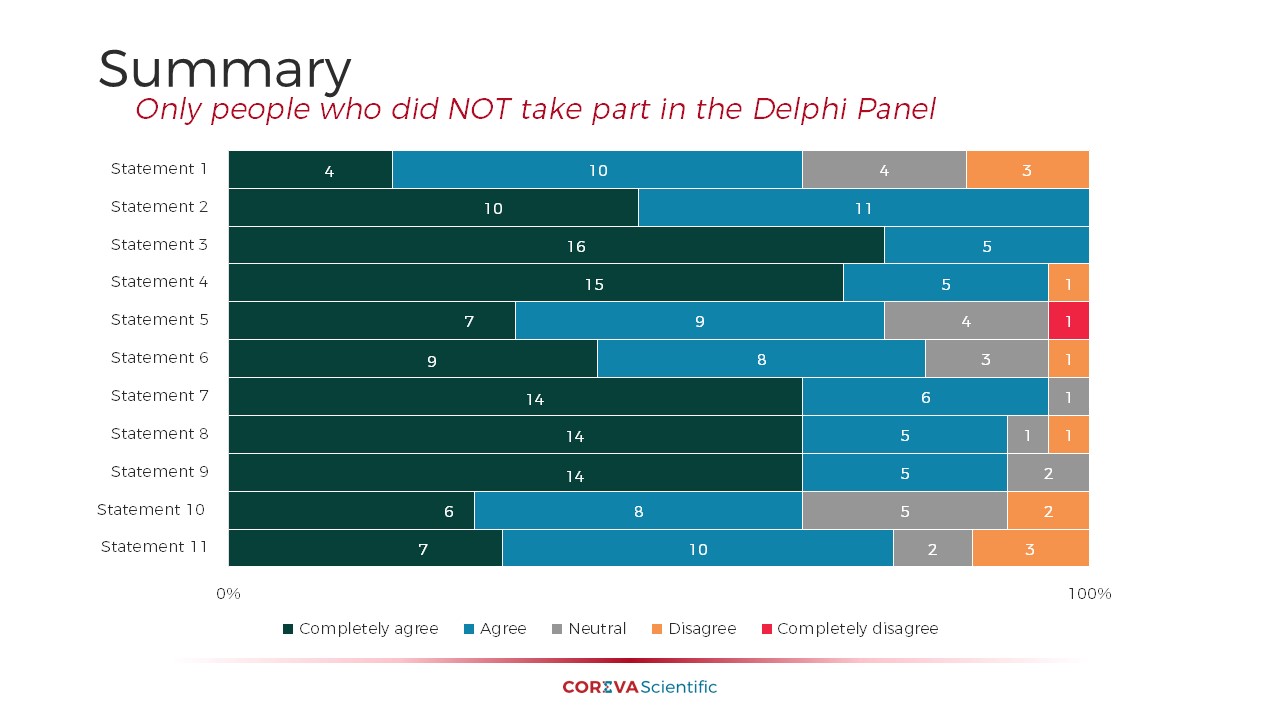

Supplement: Supplementary Data 1 [file mmc1.docx]
